# Selective β2-Adrenoceptor Blockade Rescues Mandibular Growth Retardation in Adolescent Rats Exposed to Chronic Intermittent Hypoxia

**DOI:** 10.3389/fphys.2021.676270

**Published:** 2021-06-17

**Authors:** Haixin Hong, Jun Hosomichi, Hideyuki Maeda, Yuji Ishida, Risa Usumi-Fujita, Ken-ichi Yoshida, Takashi Ono

**Affiliations:** ^1^Department of Orthodontic Science, Graduate School of Medical and Dental Sciences, Tokyo Medical and Dental University, Tokyo, Japan; ^2^Department of Forensic Medicine, Graduate School of Medicine, Tokyo Medical University, Tokyo, Japan; ^3^Department of Stomatology, Shenzhen University General Hospital, Shenzhen, China

**Keywords:** obstructive sleep apnea, intermittent hypoxia, β2-adrenergic receptor, leptin, serotonin, skeletal growth, RANKL

## Abstract

Activation of the sympathoadrenal system is associated with sleep apnea-related symptoms and metabolic dysfunction induced by chronic intermittent hypoxia (IH). IH can induce hormonal imbalances and growth retardation of the craniofacial bones. However, the relationship between IH and β2-adrenergic receptor signaling in the context of skeletal growth regulation is unclear. This study aimed to investigate the role of β2-adrenergic receptors in IH-induced mandibular growth retardation and bone metabolic alterations. Male 7-week-old Sprague–Dawley rats were subjected to IH for 3 weeks. IH conditions were established using original customized hypoxic chambers; IH was induced at a rate of 20 cycles per hour (oxygen levels changed from 4 to 21% in one cycle) for 8 h per day during the 12 h “lights on” period. The rats received intraperitoneal administration of a β2-adrenergic antagonist (butoxamine) or saline. To exclude dietary effects on general growth, the normoxic rats with saline, normoxic rats with butoxamine, and IH rats with butoxamine were subjected to food restriction to match the body weight gains between IH and other three groups. Body weight, heart rate, blood pressure, and plasma concentrations of leptin, serotonin, and growth hormone were measured. Bone growth and metabolism were evaluated using radiography, microcomputed tomography, and immunohistochemical staining. Plasma leptin levels were significantly increased, whereas that of serotonin and growth hormone were significantly decreased following IH exposure. Leptin levels recovered following butoxamine administration. Butoxamine rescued IH-induced mandibular growth retardation, with alterations in bone mineral density at the condylar head of the mandible. Immunohistochemical analysis revealed significantly lower expression levels of receptor activator of nuclear factor-kappa B ligand (RANKL) in the condylar head of IH-exposed rats. Conversely, recovery of RANKL expression was observed in IH-exposed rats administered with butoxamine. Collectively, our findings suggest that the activation of β2-adrenergic receptors and leptin signaling during growth may be involved in IH-induced skeletal growth retardation of the mandible, which may be mediated by concomitant changes in RANKL expression at the growing condyle.

## Introduction

Intermittent hypoxia (IH) during sleep is a prominent feature in both adults and pediatric patients with obstructive sleep apnea (OSA). Pediatric OSA is a risk factor for cardiovascular (Urschitz et al., 2003; Azagra-Calero et al., 2012; Ingram et al., 2017), metabolic, and neurocognitive complications during childhood (Lal et al., 2012), as well as for the onset of compromised immunosurveillance (Cubillos-Zapata et al., 2020) and OSA in adulthood (Marcus et al., 2012). Growth failure and growth retardation of the craniofacial bones are considered a symptom and consequence, respectively, of pediatric OSA (Balbani et al., 2005; Huang and Guilleminault, 2012).

Chronic IH exposure alters bone metabolism in the mandibular bone and is associated with increased *VEGF* gene expression in adolescent rats (Oishi et al., 2016a). IH-induced growth deficiency in the craniofacial and mandibular bones with dentofacial morphologic discrepancies are observed in young rats (Oishi et al., 2016b; Hosomichi et al., 2017). Additionally, chronic IH induces narrowing of the nasal cavities (Kuma et al., 2014) and turbinate hypertrophy via upregulation of genes associated with inflammatory markers in the nasal mucosa, including tumor necrosis factor (TNF)-α, interleukin (IL)-1β, and inducible nitric oxide synthase; and epithelial-mesenchymal transition markers such as transforming growth factor-beta 1, collagen I, and periostin (Kuma et al., 2020). These factors may exacerbate respiratory insufficiency in a vicious cycle. IH exposure, in human and animal models, is known to induce prolonged sympathoadrenal activation, which contributes to a constellation of symptoms, including pulmonary arterial hypertension, myocardial dysfunction, and inflammation (Nagai et al., 2014a). Nevertheless, the role of adrenergic receptors in craniofacial bone metabolism under IH is unknown.

IH exposure increases leptin levels in the liver of neonatal rats (Cai et al., 2018). Leptin inhibits bone formation by activating the sympathetic nervous system, and β-adrenergic receptor activation impedes osteoblast formation and promotes osteoclast differentiation, leading to the loss of bone mass (Takeda et al., 2002). β2-adrenergic receptors are predominantly expressed in the bone cells, whereas only low levels of β1- and β3-receptors are detectable (Aitken et al., 2009). One study reported that the expression of β2-adrenergic receptors was significantly low in leptin receptor-deficient rats, which exhibit higher bone mass (Yamamoto and Kosaka, 2009). These data imply that IH induces activation of β2-adrenergic receptors by increasing leptin levels.

Bone formation and development are critically regulated by receptor activator of nuclear factor-kappa B ligand (RANKL) and its decoy receptor, osteoprotegerin (OPG). Noradrenaline and selective β2-adrenergic agonists stimulate osteoclast formation and differentiation either directly or indirectly via increased expression of RANKL in osteoblasts, without affecting osteoblast differentiation and function (Aitken et al., 2009). Moreover, administration of a low-dose selective β2-adrenergic antagonist, butoxamine, reduces differentiation of osteoclast and increases osteoblast activity in spontaneously hypertensive rats (Arai et al., 2013). However, the involvement of β2-adrenergic receptors in the RANKL/OPG axis in the context of chronic IH-associated bone growth retardation remains unclear.

Decreased secretion of the neurotransmitter, serotonin, was reported to be observed in rats exposed to 1 week of IH (Ling et al., 2001). Serotonin level in the brain positively regulates bone mass, whereas peripheral serotonin directly inhibits bone formation via serotonin receptors in osteoblasts (Ducy, 2011). Furthermore, IH has been reported to impair the hepatic signaling of growth hormone (GH) and insulin-like growth factor I (IGF-I), leading to physical growth retardation in neonatal rats (Cai et al., 2018). Thus, changes in serotonin and GH secretion may underpin IH-induced mandibular growth retardation, and a comparison of plasma concentrations of serotonin and GH between IH-exposed and control groups would provide crucial mechanistic insights.

This study aimed to investigate the involvement of β2-adrenergic receptor signaling in IH-induced bone growth deficits of the mandible and bone metabolic alterations. We hypothesized that the leptin–serotonin–β2-adrenergic receptor axis would be implicated in mandibular bony growth retardation via alterations in IH-induced RANKL expression. To test our hypothesis, we performed an interventional study in adolescent rats subjected to chronic IH and a treated with a β2-adrenergic receptor antagonist (β2A).

## Materials and Methods

### Ethics

The experimental procedures used in this study were approved by the Institutional Animal Care and Use Committee of Tokyo Medical University (ethics approval numbers: R1-0125 and R2-0035).

### Experimental Model

**FIGURE 1:**
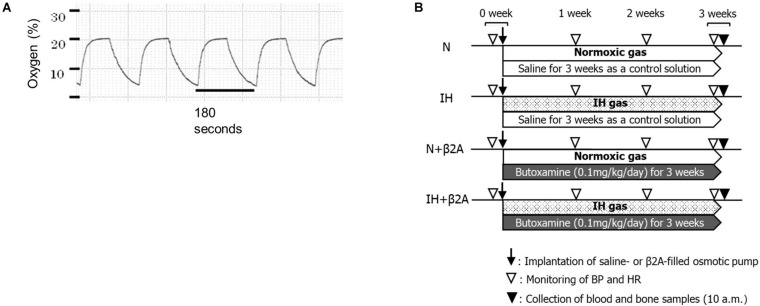
**(A)** Changes in oxygen levels in the IH chamber during the IH cycles and **(B)** experimental timeline. β2A, β2-adrenergic receptor antagonist; BP, blood pressure; HR, heart rate; IH, intermittent hypoxia; N, normoxic gas.

Experiments were conducted on 7-week-old male Sprague–Dawley rats. IH conditions were established using original, customized hypoxic chambers; IH was induced at a rate of 20 cycles per hour (nadir, 4% oxygen; peak, 21% oxygen; 0% carbon dioxide) for 8 h per day during the 12 h “lights-on” period (Figure 1A). The rats were exposed to IH or room air (normoxia; N) for 3 weeks in the same plastic cages equipped with the IH apparatus. Exposure to the original hypoxic chamber leads to severe and reproducible hypoxemia, hypocapnia, and acute respiratory alkalosis in adolescent IH-induced rats, as described elsewhere (Nagai et al., 2014b).

The IH-exposed and control rats were intraperitoneally administered with either a selective β2A (butoxamine, 0.1 mg/kg/day; Sigma-Aldrich Co., St. Louis, MO, United States) or saline, slowly released from an ionic pump (Osmotic minipump; Muromachi Kikai Co., Ltd., Tokyo, Japan) throughout the entire 3-week experimental period. Each rat was given a number randomly by simple randomization before the experiment. The number was marked on the tail of each rat. Then the rats were allocated into four experimental groups. Number 1–11 were allocated into normoxic rats administered with saline (N group, *n* = 11). Number 12–22 were allocated into IH-exposed rats administered with saline (IH group, *n* = 11). Number 23–33 were allocated into IH-exposed rats administered with butoxamine (IH + β2A group, *n* = 11), and number 34 to 44 were allocated into normoxic rats administered with butoxamine (N + β2A group, *n* = 11). All experimental groups were investigated from 7 to 10 weeks of age. During this period, the rats undergo significant growth and development of the maxillofacial bones (Hong et al., 2020). In our pilot study, we observed that IH decreased appetite in rats, resulting in a significant decrease in body weight in IH group unless food restriction was applied to the normoxic group (including both N and N + β2A groups). Therefore, the normoxic groups (N and N + β2A groups) and the IH + β2A groups were food-restricted to ensure that body weights matched between the IH and other three groups at baseline to exclude dietary effects on general growth. We monitored the amount of food assumption and body weight of all groups every day throughout the experimental period. Water was available *ad libitum.* Body weights were measured once every 2 days. After 3 weeks, the rats were anesthetized with isoflurane and euthanized. Monitoring and measurements were conducted during or after the IH exposure period (Figure 1B).

### Evaluation of Cardiovascular Parameters Over Time

Heart rate and blood pressure were measured before the experiment and once a week during the experiment. For baseline measurements, the heart rate and blood pressure before IH exposure were compared with the values measured at the first, second, and third week of the IH exposure period. Heart rate and blood pressure, including systolic blood pressure, diastolic blood pressure (DBP), and mean blood pressure (MBP), were monitored using a tail-cuff (BP-98A-L; Softlon Co., Tokyo, Japan). Body temperature was maintained at 36–38°C with a heat-retaining cylinder (THC-31; Softlon Co., Tokyo, Japan). Heart rate and blood pressure were measured thrice per rat, and the values were averaged.

### Enzyme-Linked Immunosorbent Assay (ELISA) for Leptin, Serotonin, and GH in Plasma

Plasma samples were collected from rats when they were being sacrificed at approximately 10 a.m. The rats have been exposed to IH until immediately before being sacrificed. The samples were diluted using a dilution buffer. The dilution factor was selected based on the range of the standard curve obtained using the kit standards and expected initial plasma concentrations. Rat leptin ELISA kit (Abcam, Cambridge, United Kingdom), rat serotonin ELISA kit (Novus Biologicals, Centennial, CO, United States), and rat GH ELISA kit (Shibayagi Co., Ltd., Gunma, Japan) were used for quantitative analysis of plasma levels of serotonin, leptin, and GH, respectively.

### Radiographic Analysis

**FIGURE 2:**
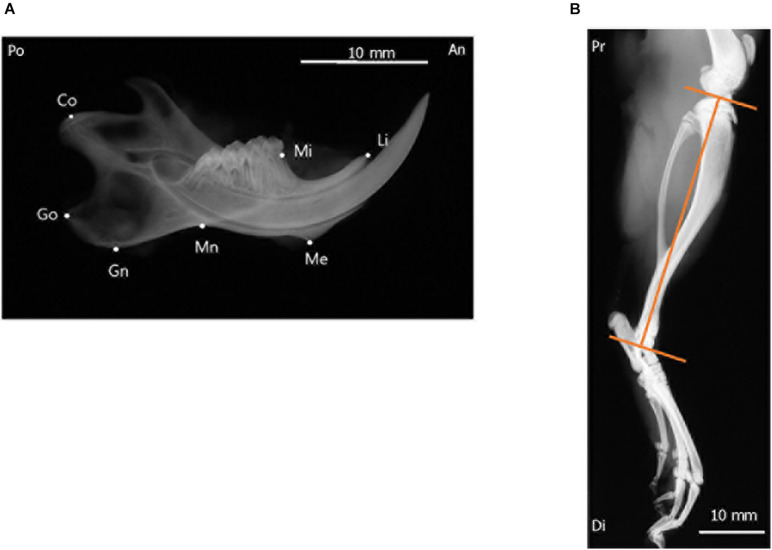
Radiographic landmarks for measurements of the craniofacial and tibial bones. **(A)** Lateral radiograph of the separated hemi-left mandible. **(B)** Radiograph of the tibia of a 10-week-old rat. Images were obtained from the control group and are shown for illustrative purposes. An, anterior; Di, distal; Po, posterior; Pr, proximal.

To evaluate mandibular growth, lateral radiographs of the separated hemi-mandible of each rat (Hichijo et al., 2014) was obtained with a soft X-ray machine (SOFTEX CMB-2; SOFTEX Co., Ltd., Tokyo, Japan). The settings used for lateral radiography of the separated mandible were as follows: tube voltage, 30 kVp; tube current, 15 mA; and 10 s impulses. All radiographs were acquired, developed, and scanned by the same operator. The operator was blinded to experimental groups. Seven landmarks were identified in the mandibular radiographs, and six linear distances between the landmarks (Hichijo et al., 2014) were measured (Figure 2A and Table 1). To ensure reliability and repeatability of the measurements, each distance was measured thrice by the same observer, and the values were averaged for analysis. To test intra-examiner reliability, three of the samples were randomly selected for measurement by the same examiner, and the intraclass correlation coefficient value was measured. Tibial length was measured from the proximal end to the distal end of the tibia in the radiographs (Figure 2B), and was used as an index for evaluating whole-body growth.

**TABLE 1:** Definitions of landmarks and variables in the mandible.

Landmarks	
Co	The most posterior and superior points on the mandibular condyle
Go	The most posterior point on the mandibular ramus
Mn	The most concave portion of the concavity on the inferior border of the mandibular corpus
Gn	The most inferior point on the ramus that lies on a perpendicular bisector of the Go-Mn line
Me	The most inferior and anterior points on the lower border of the mandible
Li	The most anterior and superior points on the alveolar bone of the mandibular incisor
Mi	The junction of the alveolar bone and mesial surface of the first mandibular molar
**Linear measurements**	
Co-LI	Total mandibular length
Co-Me	Length from the condylar head to the menton
Co-Mn	Condylar length
Co-Gn	Ramus height
Go-Mn	Posterior corpus length
Mi-Li	Anterior corpus length

### Three-Dimensional Microcomputed Tomography Analysis of the Mandible

**FIGURE 3:**
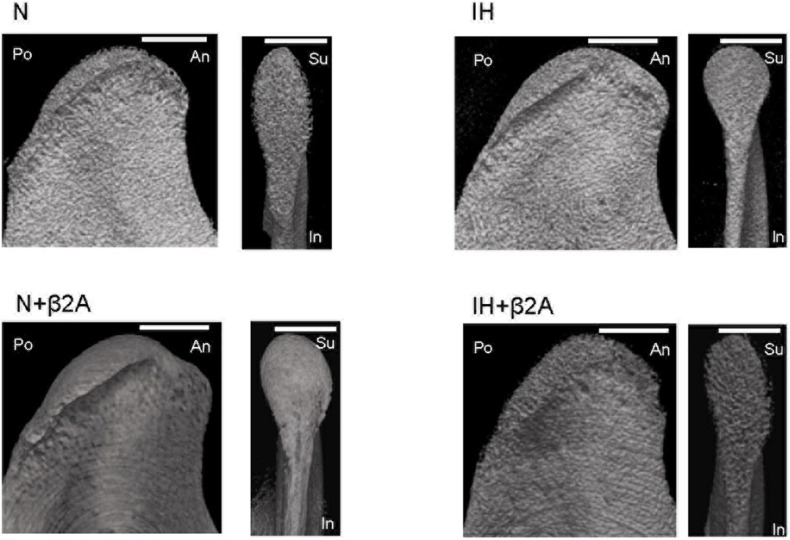
Representative sagittal and frontal images of the mandibular condyle in the four groups. Scale bar: 70 μm. β2A, β2-adrenergic receptor antagonist; An, anterior; IH, intermittent hypoxia; In, inferior; N, normoxic gas; Po, posterior; Su, superior.

The cancellous bone in the rat condylar head was analyzed using a microcomputed tomography (micro-CT) system (SMX-100CT; Shimadzu, Kyoto, Japan) to evaluate bone microstructure and bone mineral density (BMD). The region of interest for structural morphometry analysis was selected based on previous studies (Shimizu et al., 2011; Oishi et al., 2016b). Three-dimensional images of the condylar head of the animals in all four experimental groups were reconstructed using micro-CT (Figure 3).

### Histochemical Analysis

The bones used for micro-CT analysis (i.e., the mandibular condylar head) were fixed in 4% paraformaldehyde and then decalcified in 10% EDTA for 6 weeks. The decalcified samples were then embedded in paraffin, and 2.5 μm-thick longitudinal sections were obtained. After deparaffinization, the sections were stained with toluidine blue to observe histomorphological changes. To perform immunohistochemical analysis, after deparaffinization and blocking of endogenous peroxidase activity with 0.3% H_2_O_2_ in methanol, the sections were heated in 0.01 M citrate buffer (pH 6.0) for antigen retrieval at 121°C for 10 min. VECTASTAIN Elite ABC-horseradish peroxidase (HRP) R.T.U. Kit (Vector Laboratories, Burlingame, CA, United States) was used, which included normal horse plasma, secondary antibody and VECTASTAIN Elite R.T.U. ABC Reagent. Non-specific antibody binding was blocked by incubating the tissue sections with normal horse plasma for 20 min at room temperature. The sections were then incubated overnight at 4°C with the following primary antibodies: anti-TRANCE/TNFSF11/RANKL antibody (1:800 dilution; Novus Biologicals, Centennial, CO, United States) and anti-OPG/TNFRSF11B antibody (1:800 dilution; Novus Biologicals). The sections were then incubated in prediluted biotinylated secondary antibody for 30 min at room temperature, and then in VECTASTAIN Elite R.T.U. ABC Reagent for another 30 min at room temperature. Next, the sections were incubated in HRP substrate solution for 1 min. Finally, the sections were counterstained with hematoxylin. Phosphate-buffered saline containing 1% bovine plasma albumin was used as the isotypic control. Sections of rat lung tissue and liver tissue were used as positive controls. An 16 × 73 μm field from the center of the middle one-third of the longitudinal condylar head was selected as a region of interest (ROI) and subjected to quantitative analysis using Image J software (National Institutes of Health, United States). The same ROI was selected for each sample. The number of positive cells in the ROI was quantified as a proxy of tissue protein expression levels.

### Statistical Analyses

All data are reported as mean ± standard deviation. Statistical analyses were performed using IBM SPSS Statistics version 22.0 (Chicago, IL, United States). A test for homogeneity of variances was first conducted. For groups with equivalent variances, analysis of variance with Dunnett’s *t*-test was conducted to compare the mean values between the experimental and control groups. When variances were not equivalent, Dunnett’s T3 test (Lee and Lee, 2018) was conducted to compare the mean values. Corrections for multiple comparisons were applied for one-way analysis of variance and Dunnett’s *t*-test or Dunnett’s T3 test depending on the results of the test for homogeneity of variances. A *p* < 0.05 was considered statistically significant.

## Results

### General Growth

To assess the effects of IH and β2A on mandibular bone metabolism, the rats of the N, N + β2A and IH + β2A groups were subjected to food restriction to match the body weight gains between the N and IH groups, in order to avoid diet-induced alterations in bone turnover. Approximately 16 g food per day was given to each rat in the N, N + β2A and IH + β2A groups, which was determined in reference to the daily average amount of food consumption of the IH group. No significant differences in body weight were observed between the four groups (Table 2). We observed a significant decrease in tibial length (Table 2) in the IH group (36.93 ± 0.64 mm) when compared with that in the N group (38.02 ± 0.68 mm) [*F*(3, 40) = 3.881, *p* = 0.014]. Butoxamine administration resulted in the recovery from IH exposure-induced tibial growth retardation [38.01 ± 0.86 mm in the IH + β2A group vs. 38.02 ± 0.68 mm in the N group, *F*(3, 40) = 3.881, *p* = 0.996].

**TABLE 2:** Body weight (grams), body weight gain rate (%), and tibial length (mm) of rats at 3 weeks after IH exposure.

	N group	IH group	N + β2A group	IH + β2A group
Body weight (g)	248.27 ± 25.26	239.05 ± 16.85	261.86 ± 14.86	258.32 ± 17.81
Body weight gain rate (%)	9.61 ± 11.58	4.08 ± 5.40	15.07 ± 8.56	11.77 ± 8.24
Tibial length (mm)	38.02 ± 0.68	36.93 ± 0.64*	37.41 ± 1.24	38.01 ± 0.86

Values are presented as mean ± standard deviation.N, rats exposed to normoxic gas; IH, rats exposed to intermittent hypoxia; N + β2A, rats exposed to normoxic gas and butoxamine medication; IH + β2A, rats exposed to IH and butoxamine medication.n = 11 per group. *P < 0.05 vs. N group.

### Heart Rate and Blood Pressure Changes Induced by IH

**FIGURE 4:**
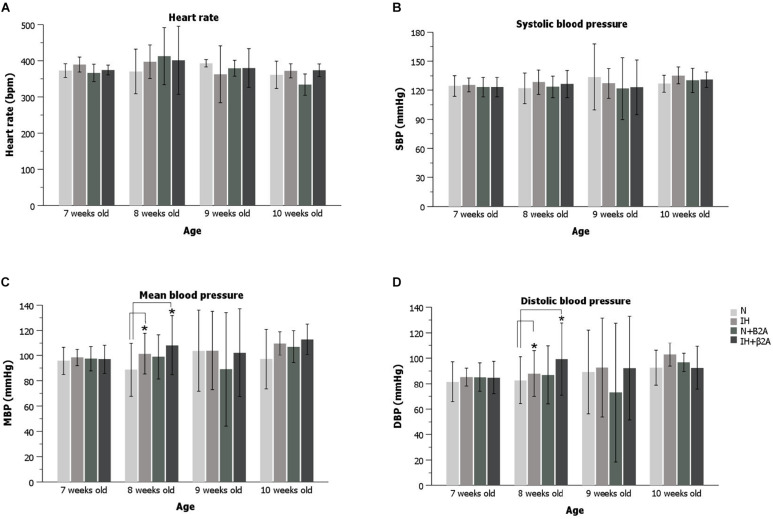
Heart rate and blood pressure of rats for the 3-week experiment period. **(A)** Heart rate changes during the experiment; **(B)** SBP changes during the experiment; **(C)** MBP changes during the experiment; and **(D)** DBP changes during the experiment. *n* = 11 per group. *P < 0.05, one-way ANOVA with two-tailed Dunnett’s *t*-test for **(A–C)**; one-way ANOVA with Dunnett’s T3 test for **(D)**. ANOVA, analysis of variance; SBP, systolic blood pressure; DBP, diastolic blood pressure; MBP, mean blood pressure.

Significant increases were observed in both the MBP (Figure 4C) and DBP (Figure 4D) of rats after 1 week of IH exposure [MBP: 108.1 ± 5.9 mmHg in the IH group vs. 89.1 ± 15.7 mmHg in the N group, *F*(3, 40) = 5.320, *p* = 0.011; DBP: 93.4 ± 7.4 mmHg in the IH group vs. 75.2 ± 13.9 mmHg in the N group, *F*(3, 40) = 7.532, *p* = 0.003]. Butoxamine administration in conjunction with IH exposure resulted in a significant increase in MBP (Figure 4C) and DBP (Figure 4D) in 8-week-old rats (MBP: 110.0 ± 12.8 mmHg in the IH + β2A group vs. 89.1 ± 15.7 mmHg in the N group, *F*(3, 40) = 5.320, *p* = 0.002; DBP: 100.2 ± 12.9 mmHg in the IH + β2A group vs. 75.2 ± 13.9 mmHg in the N group, *F*(3, 40) = 7.532, *p* = 0.0003). However, no significant differences were apparent in the blood pressure parameters between the IH and normoxic groups after 2 and 3 weeks of IH exposure. Moreover, no significant differences were observed in heart rate and blood pressure between the N + β2A and N groups (Figures 4A–D).

### Plasma Levels of Leptin, Serotonin, and GH in IH-Exposed Rats

**FIGURE 5:**
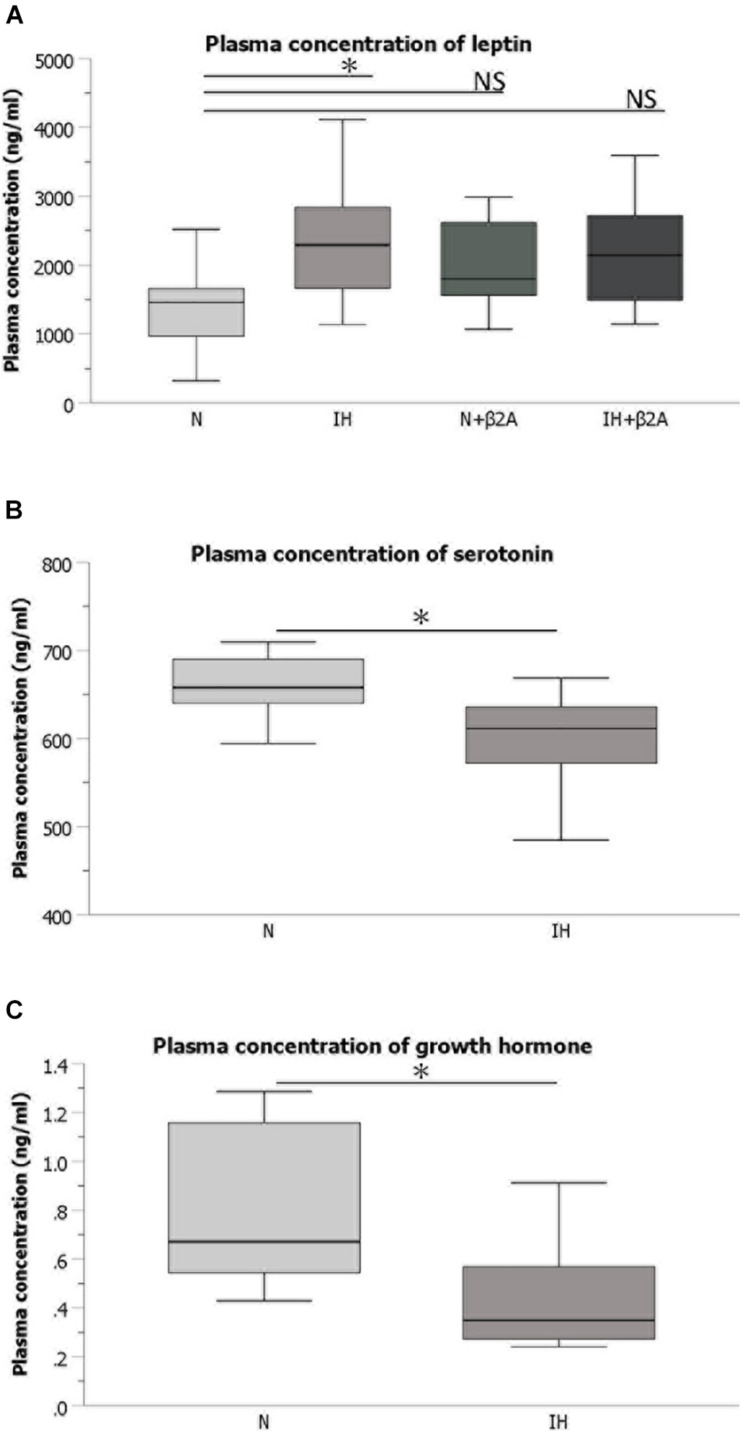
Plasma concentrations of hormones associated with bone metabolism. **(A)** Comparison of plasma concentrations of leptin among the four groups Comparison of plasma concentrations of serotonin **(B)** and growth hormone **(C)** between IH and N groups. *n* = 11 per group. ^∗^*P* < 0.05, one-way ANOVA with two-tailed Dunnett’s *t*-test for **(A)**; two-tailed *t*-test for **(B,C)**. ANOVA, analysis of variance; β2A, β2-adrenergic receptor antagonist; IH, intermittent hypoxia; N, normoxic gas; NS, not significant.

ELISA results indicated that plasma concentration of leptin were significantly increased in IH-exposed adolescent rats [2.36 ± 0.99 in the IH group vs. 1.37 ± 0.65 in the N group, *F*(3, 40) = 2.738, *p* = 0.03] (Figure 5A), whereas a significant decrease was observed in the plasma levels of serotonin [604.59 ± 51.07 in the IH group vs. 660.29 ± 32.61 in the N group, *F*(3, 40) = 31.175, *p* = 0.028] (Figure 5B) and GH [0.44 ± 0.23 in the IH group vs. 0.81 ± 0.34 in the N group, *F*(3, 40) = 3.961, *p* = 0.027] (Figure 5C). Butoxamine administration resulted in the normalization of the high leptin level induced by IH exposure (Figure 5A). These results indicate that IH negatively impacts hormones related to bone growth and that the impairment in leptin level was rescued by β2A.

### Effects of β2-Adrenergic Antagonist Administration on Impaired Mandibular Growth in Ih-Exposed Rats

**FIGURE 6:**
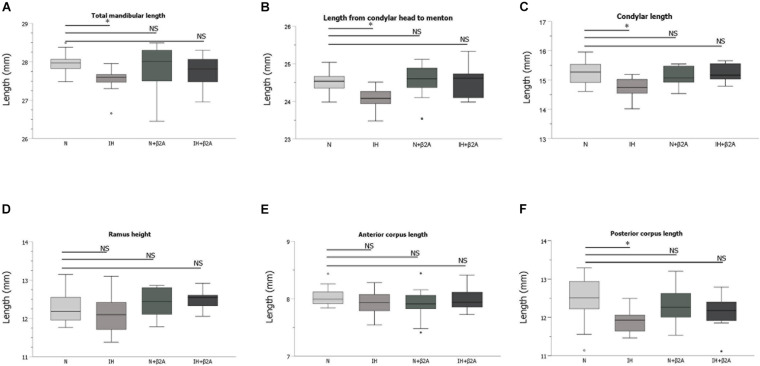
Linear measurement results of the mandible after 3-week IH exposure. **(A–F)** Comparison of changes in skeletal growth on the lateral cephalograms between four groups after 3-week IH exposure. *n* = 11 per group. ^∗^*P* < 0.05, one-way ANOVA with two-tailed Dunnett’s *t*-test. ANOVA, analysis of variance; β2A, β2-adrenergic receptor antagonist; IH, intermittent hypoxia; N, normoxic gas; NS, not significant.

Radiographic analyses of the mandible revealed that the total mandibular length (Co-Li) (Figure 6A), length from the condylar head to menton (Co-Me) (Figure 6B), condylar length (Co-Mn) (Figure 6C), and posterior corpus length (Go-Mn) (Figure 6F) in the IH group were significantly shorter than those in the N group [Co-Li: 27.5 ± 0.3 mm in the IH group vs. 28.0 ± 0.2 mm in the N group, *F*(3, 40) = 1.853, *p* = 0.016; Co-Me: 24.1 ± 0.3 mm in the IH group vs. 24.5 ± 0.3 mm in the N group, *F*(3, 40) = 3.678, *p* = 0.044; Co-Mn: 14.8 ± 0.4 mm in the IH group vs. 15.2 ± 0.5 mm in the N group, *F*(3, 40) = 4.354, *p* = 0.011; Go-Mn: 11.9 ± 0.3 mm in the IH group vs. 12.5 ± 0.7 mm in the N group, *F*(3, 40) = 2.967, *p* = 0.025]. However, these parameters were comparable between the IH + β2A and N groups (Figures 6A–F). No significant effects of butoxamine administration on mandibular growth were observed in the normoxic rats.

### BMD in the Mandibular Condyle

**FIGURE 7:**
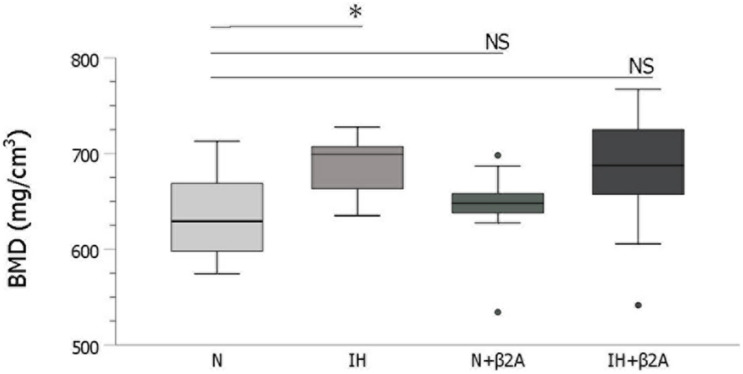
IH-induced changes in bone mineral density in the mandibular condylar head. *n* = 11 per group. ^∗^*P* < 0.05, one-way ANOVA with two-tailed Dunnett’s *t*-test. β2A, β2-adrenergic receptor antagonist; IH, intermittent hypoxia; N, normoxic gas; NS, not significant.

Based on micro-CT analysis, BMD in the condylar head was significantly higher in the IH group than in the N group [687.0 ± 32.7 mm in the IH group vs. 632.9 ± 44.3 mm in the N group, *F*(3, 40) = 2.508, *p* = 0.047] (Figure 7). BMD was comparable between the IH + β2A and N groups.

### Expression of RANKL and OPG in the Mandibular Condyle in IH-Exposed Rats

**FIGURE 8:**
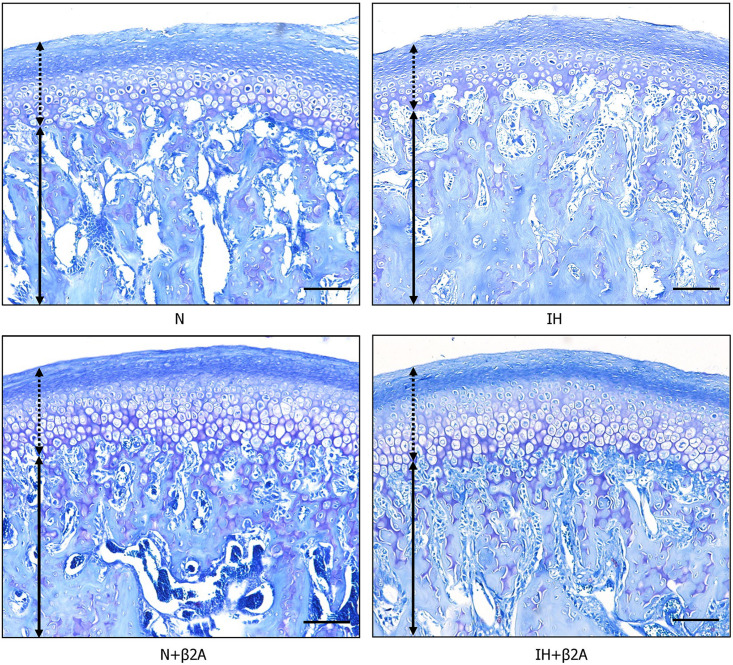
Images of toluidine blue staining in the mandibular condylar head after IH and administration of butoxamine. Arrows indicate mandibular cartilage and dotted arrows indicate subchondral bone. Magnification: 10×. Scale bar: 100 μm. β2A, β2-adrenergic receptor antagonist; IH, intermittent hypoxia; N, normoxic gas.

Toluidine blue staining showed thinner cartilage in the mandibular condyle of the IH group (Figure 8). Moreover, larger subchondral bone marrow cavities were observed in the N group, compared to the IH group.

**FIGURE 9:**
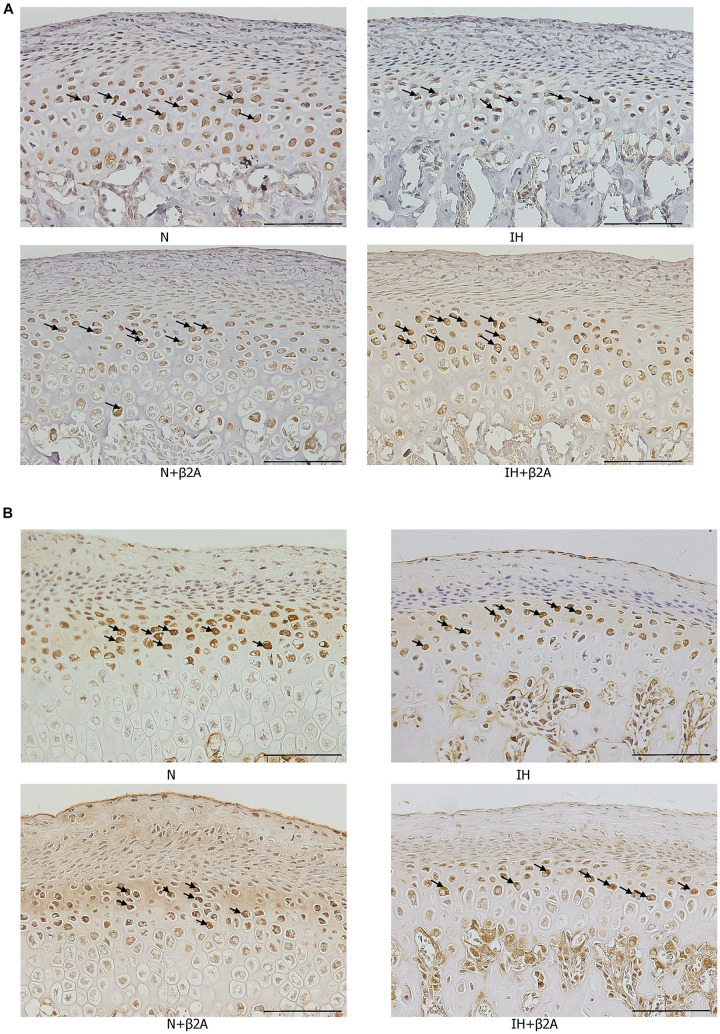
Images of immunohistochemical staining for RANKL and OPG in the mandibular condylar head after IH and administration of butoxamine. Representative images of immunostainings for RANKL **(A)** and OPG **(B)** in the mandibular condylar cartilage in the control and three experimental groups. Arrows indicate immuno-positive cells. Magnification: 20×. Scale bar: 100 μm. β2A, β2-adrenergic receptor antagonist; IH, intermittent hypoxia; OPG, osteoprotegerin; RANKL, receptor activator of nuclear factor-kappa B ligand; N, normoxic gas.

RANKL- and OPG-positive cells in the growing mandibular condyle were detected using immunohistochemistry to investigate whether the effects of IH and β2-adrenergic receptor signaling on bone growth were mediated via changes in RANKL/OPG equilibrium. Immunohistochemical staining revealed that the number of RANKL-positive cells in the condylar head was significantly decreased in the IH group, whereas recovery was observed in the IH + β2A group (Figure 9A and Table 3). In contrast, no significant differences were noted in the number of OPG-positive cells among the four groups (Figure 9B and Table 3).

**TABLE 3:** Cell count per 1,168 μm^2^ of immuno-positive cells for RANKL and OPG in the condylar head.

	*N*	IH	N + β2A	IH + β2A
RANKL	39.0 ± 7.8	32.3 ± 8.3	43.2 ± 3.3	35.9 ± 8.4
OPG	38.0 ± 6.6	36.2 ± 5.1	38.0 ± 7.8	39.0 ± 3.6

Values are presented as mean ± standard deviation.RANKL, receptor activator of nuclear factor-kappa B ligand; OPG, osteoprotegerin; N, rats exposed to normoxic gas; IH, rats exposed to intermittent hypoxia; N + β2A, rats exposed to normoxic gas and butoxamine medication; IH + β2A, rats exposed to IH and butoxamine medication.n = 11 per group. *P < 0.05 vs. N group.

## Discussion

Herein, we present novel evidence of IH-induced growth retardation of the mandibular bone via activation of β2-adrenergic receptors, which was rescued by β2A administration. In rats exposed to IH, bone growth retardation and alterations in RANKL expression were observed in the mandibular condyle, which is the center of the most substantial growth in the craniofacial complex. IH resulted in systemic changes, including slow tibial growth, increased secretion of leptin, and decreased secretion of serotonin and GH. β2-receptors in the bone cells are a target of the leptin–serotonin axis (Takeda et al., 2002; Elefteriou et al., 2005). Our findings indicate that impairments in the leptin–serotonin–β2 receptor axis underlies growth retardation of the mandibular bone in rats exposed to chronic IH.

ELISA results revealed that plasma leptin concentrations were significantly increased following IH exposure, and that this effect was rescued by butoxamine administration. A previous study (Berger and Polotsky, 2018) reported that IH is a potent promoter of leptin expression and release from adipose tissue, which may lead to increased leptin concentration and leptin resistance during IH exposure. This may, at least partly, underpin the observation of significantly higher plasma leptin concentrations in the IH group than that in the N group noted in this study. Moreover, other studies (Takeda et al., 2002; Elefteriou et al., 2004) have reported that leptin is a regulator of bone formation, and that this regulatory function is enacted via the sympathetic nervous system. This suggests that excessive plasma leptin levels retard bone formation via activation of the sympathetic nervous system. This mechanism may underpin the lowering of the increased plasma leptin concentrations by β2A administration, which blocked the activation of β2-receptors and rescued mandibular and tibial growth retardation.

Serotonin is a target of leptin, and leptin-dependent neuronal control of bone mass is regulated via serotonin and downstream β2-adrenergic receptors (Yadav et al., 2009). Chronic IH exposure downregulates serotonin expression in the central nervous system (Ling et al., 2001), which may directly or indirectly regulate bone metabolism (Ducy, 2011). In our study, IH exposure resulted in a significant decrease in plasma serotonin concentrations. Serotonin induces GH secretion via the release of hypothalamic GH-releasing hormone in young rats treated for upper airway obstruction (Ducy, 2011), and GH stimulates the production of IGF-1, a mediator for bone and cartilage growth (Yakar and Isaksson, 2016). The rise in plasma GH/IGF-1 levels is accompanied by peak bone acquisition during pubertal growth due to the activation of an array of genes that mediate cellular differentiation and function; conversely, their expression levels decline and induce bone loss during aging (Yakar and Isaksson, 2016). Further, administration of GH enhances mandibular growth, particularly growth in the condylar head length (Khan et al., 2013). The significant decrease in plasma GH levels in adolescent IH-exposed rats observed in our study may at least partly underlie the mandibular growth retardation, especially in the mandibular condylar head. Epitácio-Pereira et al. (2013) reported that isolated GH deficiency reduced the size but not the density of the trabecular and mixed bones. Indeed, the impact of GH deficiency on bone differs across different age groups in humans; GH deficiency predominantly causes delayed growth in children and low bone mass in adults (Tritos and Klibanski, 2016). As pair-feeding was conducted to eliminate diet-induced effects on bone turnover in growing IH-exposed rats, no significant differences in body weight were observed among the four groups. IH exposure resulted in a significant decrease in tibial length and influenced mandibular length rather than BMD in pubertal rats. This finding may explain the discrepancy between the effects of IH exposure on mandibular elongation and bone mineralization.

Previous studies (Maeda et al., 2013; Nara et al., 2015) have reported significant associations between IH and hypertension, heart failure, and other cardiovascular diseases. MBP and DBP were only found to be significantly higher after the first week of IH exposure in the IH-exposed adolescent rats than in the normoxic rats, whereas no significant differences in heart rate and systolic blood pressure were observed and the changes in MBP and DBP disappeared after 2 and 3 weeks of IH exposure. Administration of low-dose butoxamine did not significantly impact these IH-induced changes, according to a previous study (Arai et al., 2013) that investigated the effects of butoxamine on bone metabolism in spontaneously hypertensive rats (SHRs). The authors of that study suggested that the presence of hypertension and development of osteoporosis in SHRs were not correlated. Hypertension is predominantly caused by the overactivation of α-adrenergic receptors (Taylor and Cassagnol, 2020), whereas osteoporosis is mainly induced by β2-adrenergic receptors (Elefteriou et al., 2005; Kondo et al., 2013). Thus, low-dose β2A administration may induce anti-osteoporotic effects without recovery of blood pressure. IH-induced cardioprotection during ischemia/reperfusion has been reported to result in an increase in coronary flow, improvements in coronary endothelial function, and prevention of myocardial mitochondrial Ca^2+^ overload, which protects the heart via several pathways (Mallet et al., 2018; Chang et al., 2019). Moreover, another study reported that the expression of proteins related to cardiac damage was increased in response to IH as early as 3 days, and decreased gradually over 4–8 weeks (Zhou et al., 2017). These factors may explain the automatic recovery of higher MBP and DBP observed during the first week of IH exposure after extending the duration of IH exposure to 2–3 weeks in our study.

OPG and RANKL are key molecular mediators of osteoblast–osteoclast coupling (Hsu et al., 1999; Lacey et al., 2012). RANKL binds to its receptor, RANK, on osteoclast lineage cells and promotes osteoclast differentiation, activation, and survival, thereby promoting bone resorption (Trouvin and Goëb, 2010). In contrast, OPG inhibits the activation and function of osteoclasts. Based on immunohistochemical staining results for RANKL and OPG proteins in the mandibular condyle, we observed reduced RANKL expression in the condylar head in the IH group, which could explain the microstructural changes associated with higher bone density in the condylar head in this group. Increased BMD in the mandibular (Oishi et al., 2016a) and condylar bones (Oishi et al., 2016b; Hong et al., 2020) of IH-exposed adolescent rats and in the lumbar vertebrae of IH-exposed adult rats (Guner et al., 2013) has been reported. In this regard, we observed increased BMD in the condylar head in the poorly growing mandible of IH-exposed rats. Studies using rodent models have helped to resolve the contradiction between the significantly denser microstructure of the condyle and poor growth in the endochondral bones (Takahi et al., 2002; Oishi et al., 2016a; Jing et al., 2020). Dupuis et al. demonstrated that irregular ossification induced by the retinoid X receptor agonist resulted in premature closure of the growth plate, leading to disrupted tibial growth in young rats (Dupuis et al., 2019). Takahi et al. demonstrated that overexpression of VEGF resulted in poor growth of the endochondral bones with early closure of the growth plate in a rat model of collagen-induced arthritis (Takahi et al., 2002). In addition, 3-week IH exposure, which was applied in our model, has been reported to increase BMD in the mandibular bone in association with increased *HIF1* and *VEGF* gene expression in 7-week-old rats (Oishi et al., 2016a). According to a review by Jing et al. (2020), substantial evidence supports the theory that direct cell transdifferentiation from chondrocytes to bone cells precisely connects chondrogenesis and osteogenesis in a continuous lineage-linked process of endochondral bone formation and limb elongation. Moreover, a recent histological study from our laboratory (Lekvijittada et al., 2021) demonstrated that IH reduced maturation and increased the number of hypertrophic chondrocytic layers in the middle and posterior areas of the mandibular cartilage in neonatal rats. We also demonstrated (Lekvijittada et al., 2021) that IH shifted proliferation and maturation in the mandibular condyle fibrocartilage toward hypertrophic differentiation and ossification by downregulating TGF-β and SOX9 mRNA levels, as indicated by polymerase chain reaction studies. Based on these findings, we conjecture that IH induced a decrease in the proliferative ability of condyle fibrocartilage; consequently, the morphology of the condylar head appeared more mature and the growth of the condylar head terminated earlier than that in the control group due to downregulation of RANKL protein expression—these effects collectively impacted the normal process of bone remodeling in the condylar head. This may have resulted in the shorter length and higher density of the condylar head in the IH group compared to that in the normoxic group. Furthermore, Lavine et al. reported that administration of a β2A attenuated VEGF expression in mice (Lavine et al., 2017). We observed that β2A administration reversed IH-induced changes in RANKL protein expression. In this regard, β2A administration may have altered RANKL expression via VEGF regulation in IH-exposed rats. Thus, IH exposure may have modulated RANKL protein expression by inducing the overactivation of β2-adrenergic receptors, leading to morphological and microstructural changes in the condylar head. Notably, these changes were rescued by butoxamine administration.

Collectively, our findings suggest that IH exposure shifted the RANKL/OPG equilibrium toward RANKL deficiency, resulting in microstructural changes in the subchondral bone of the mandibular condylar joint during growth. These results indicate that IH-induced retardation in bone growth via β2-adrenergic receptor signaling is implicated in RANKL expression changes in the growing condylar joint.

Several limitations of our rodent model, which simulated recurrent episodes of apneic events in pediatric OSA, should be noted. First, IH is only one of the main characteristics of OSA, and other features also contribute to OSA pathogenesis. Second, humans and rodents exhibit different bone growth patterns; hence, our findings may not be directly applicable to humans. Third, our study proposes only one potential explanation for general and local growth modifications via impaired adrenergic receptors and hormones, induced by IH. Further studies are warranted to identify the precise mechanisms underlying IH-induced mandibular growth deficits in humans.

In conclusion, our study demonstrates that the activation of β2-adrenergic receptors and leptin signaling during growth may be involved in IH-induced growth retardation of the mandible via changes in RANKL expression in the growing condyle. Furthermore, our study may bring about a new therapeutic idea on IH-induced mandibular retardation in the future.

## Data Availability Statement

The datasets generated during and/or analyzed during this study can be found in the data.bris Research Data Repository (https://data.bris.ac.uk/data/). Code will be provided upon request.

## Ethics Statement

The animal study was reviewed and approved by the Institutional Animal Care and Use Committee of Tokyo Medical University (ethics approval numbers: R1-0125 and R2-0035).

## Author Contributions

HH, JH, HM, K-iY, and TO conceived and designed the experiments. HH, JH, and HM performed the experiments. HH and JH analyzed the data and prepared the figures. HH, JH, HM, YI, RU-F, K-iY, and TO interpreted the data. HH, JH, and TO drafted and edited the manuscript. All authors approved the final version of the manuscript.

## Conflict of Interest

The authors declare that the research was conducted in the absence of any commercial or financial relationships that could be construed as a potential conflict of interest.
